# Supplementary material for: Multidrug resistance plasmids commonly reprogram the expression of metabolic genes in Escherichia coli
Source: mSystems. 2024 Feb 20;9(3):e01193-23. doi: 10.1128/msystems.01193-23 (PMC10949484; doi:10.1128/msystems.01193-23)
Supplement: Supplemenental material — Supplemental figures and tables. [file msystems.01193-23-s0003.pdf]

Supplementary

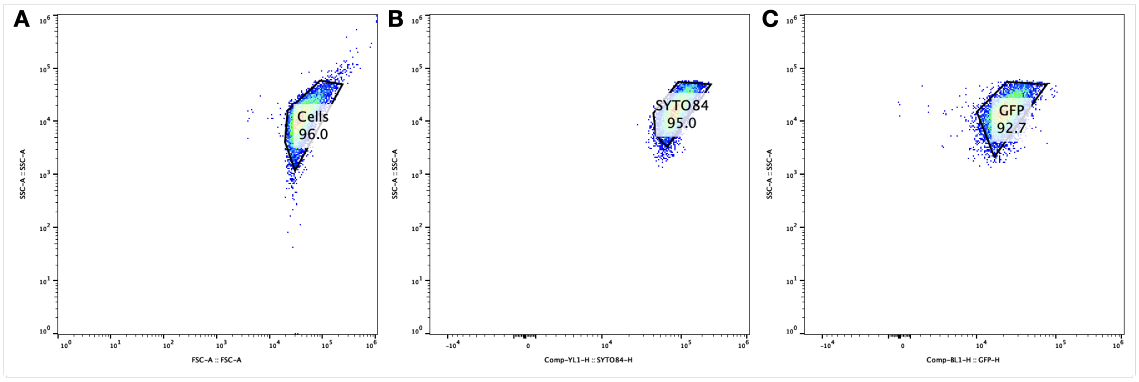

Fig. S1 Example gating applied to all test populations. **A** All cells, WT population unstained. **B** SYTO^{TM}84 stained cells. **C** GFP+ cells. Gating applied so that single (SYTO^{TM}84 only, plasmid bearer) and double (SYTO^{TM}84 stained plus GFP+ expressing, plasmid-free) labelled cells could be counted.

Table S1 Competition Index (CI) values for all independent biological replicates.

Transconjugant	Replicate	CI	Mean
	1	0.015	
7E2	2	0.036	0.032
	3	0.045	
	1	0.054	
13F2	2	0.157	0.082
	3	0.034	
	1	0.017	
LA84	2	0.005	0.012
	3	0.015	
	1	0.008	
LA232	2	0.005	0.006
	3	0.005	
	1	0.006	
LL19	2	0.006	0.005
	3	0.004	
	1	0.013	
LL34	2	0.210	0.096
	3	0.065	
	1	0.041	
LL35	2	0.024	0.025
	3	0.010	
	1	0.015	
LL70	2	0.004	0.007
	3	0.003	

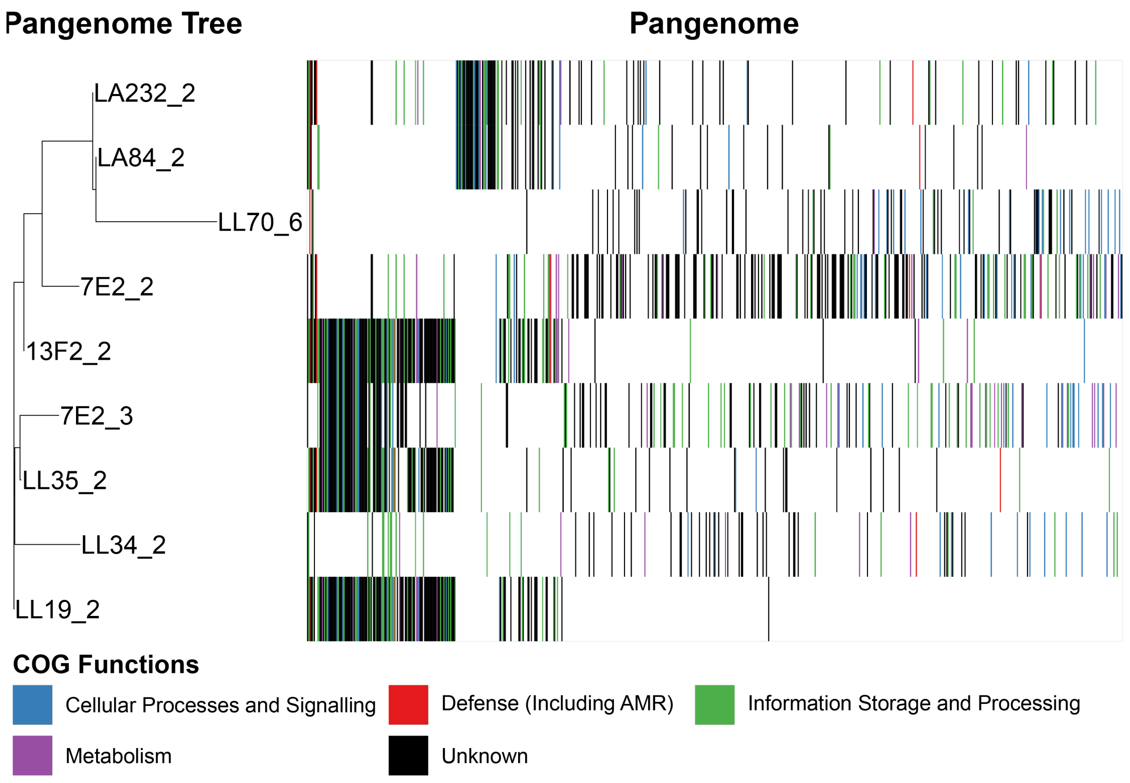

Fig. S2 Presence and absence of plasmid-encoded genes on the nine conjugated plasmids. Genes coloured by functions as assigned by eggNOG, grouped into categories for simplicity.

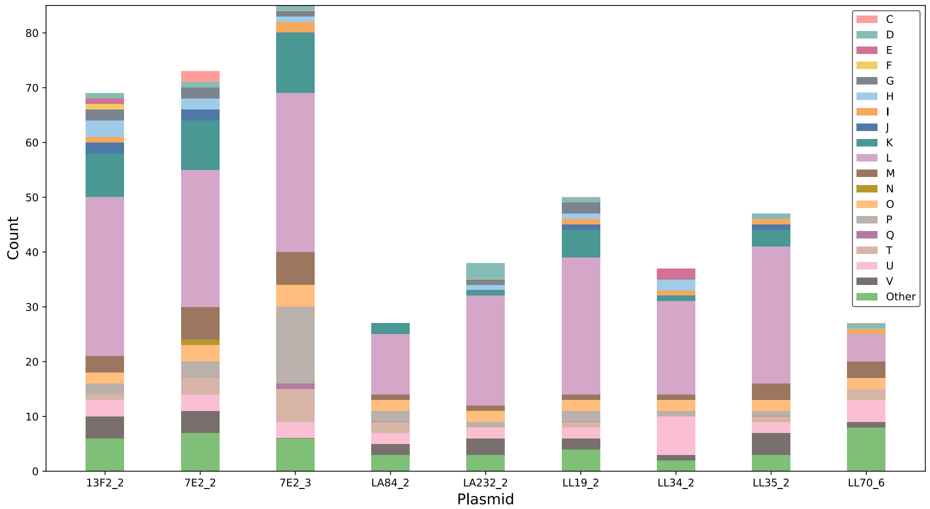

Fig. S3 COG categories of genes with known function encoded by the conjugative plasmids, as assigned by eggNOG-mapper.

Table S2 Mutations in transconjugant long read sequences as predicted by breseq. All predicted mutations were deletions. bp = base pair.

Transconjugant	Position	Deletion	Annotation	Gene	Description
7E2	3,436,101	1 bp	Coding (152/234 nt)	*ynaE* →	Rac prophage; cold shock protein, function unknown
13F2	3,436,802	1 bp	Coding (303/591 nt)	*pinR* →	Rac prophage; predicted site-specific recombinase
LA84	None				
LA232	660,566	1 bp	Intergenic (-39/+48)	*NDAJDAOJ_00649* ←/← *NDAJDAOJ_00650*	tRNA-Glu/16S ribosomal RNA
LL19	660,566	1 bp	Intergenic (-39/+48)	*NDAJDAOJ_00649* ←/← *NDAJDAOJ_00650*	tRNA-Glu/16S ribosomal RNA
LL19	3,436,802	1 bp	Coding (303/591 nt)	*pinR* →	Rac prophage; predicted site-specific recombinase
LL34	None				
LL35	None				
LL70	2,682,571	1 bp	Intergenic (+51/+32)	*yehA_2* →/← *rcnB*	Putative fimbrial protein/periplasmic protein for nickel/cobalt efflux

Table S3 Gene Ontology (GO) terms enriched across the differentially expressed from all transconjugants combined.

Ontology	GO ID	Term	Count	Expected	Fold enrichment	P value (Fisher)
Biological process	GO:0000041	Transition metal ion transport	33	14.41	2.29	1.59E-04
	GO:0006811	Monoatomic ion transport	74	41.92	1.77	2.7E-05
	GO:0006810	Transport	198	136.81	1.45	8.07E-07
	GO:0051234	Establishment of localization	199	138.31	1.44	9.09E-07
	GO:0051179	Localization	200	138.87	1.44	9.27E-07
	GO:0034220	Monoatomic ion transmembrane transport	60	34.25	1.75	2.03E-04
	GO:0055085	Transmembrane transport	182	120.72	1.51	2.33E-07
	GO:0009987	Cellular process	594	539.20	1.10	1.77E-04
	GO:0071702	Organic substance transport	133	90.58	1.47	4.74E-05
	UNCLASSIFIED	Unclassified	133	184.54	0.72	4.49E-05
	GO:0006396	RNA processing	6	22.65	0.26	1.73E-04
	GO:0043170	Macromolecule metabolic process	136	194.27	0.70	5.15E-06
	GO:0006399	tRNA metabolic process	4	18.72	0.21	1.93E-06
	GO:0034660	ncRNA metabolic process	6	26.58	0.23	9.72E-06
	GO:0051301	Cell division	2	15.16	0.13	1.83E-04
Molecular function	GO:0022857	Transmembrane transporter activity	159	103.50	1.54	7.48E-07
	GO:0005215	Transporter activity	159	105.00	1.51	1.49E-06
	GO:0140640	Catalytic activity, acting on a nucleic acid	27	56.33	0.48	4.29E-05
Cellular component	GO:1902495	Transmembrane transporter complex	82	52.97	1.55	4.50E-04
	GO:0098796	Membrane protein complex	98	63.07	1.55	1.11E-04
	GO:0016020	Membrane	350	281.86	1.24	7.08E-06
	GO:0005886	Plasma membrane	301	239.94	1.25	2.92E-05
	GO:0071944	Cell periphery	334	261.65	1.28	1.24E-06

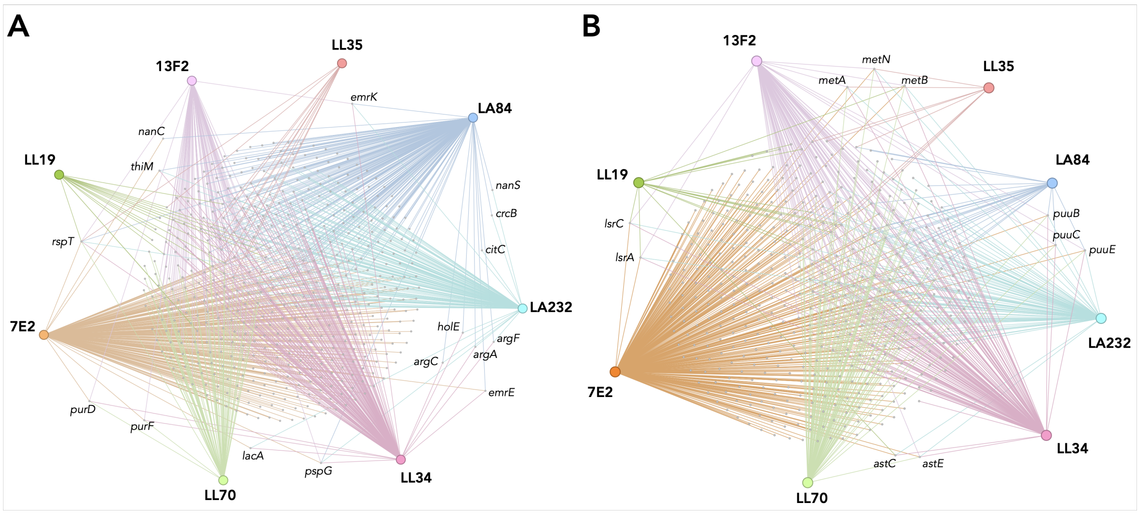

Fig. S4 Genes of known function that are **A** up- or **B** downregulated in the eight transconjugants. Nodes that depict transconjugants are labelled in bold. Edges connect the transconjugants to their significantly differentially expressed genes. Select genes are highlighted.
